# Correlates of engaging in sports and exercise volunteering among older adults in Japan

**DOI:** 10.1038/s41598-022-07688-1

**Published:** 2022-03-08

**Authors:** Taishi Tsuji, Satoru Kanamori, Mitsuya Yamakita, Ayane Sato, Meiko Yokoyama, Yasuhiro Miyaguni, Katsunori Kondo

**Affiliations:** 1 Faculty of Health and Sport Sciences, University of Tsukuba, 3-29-1 Otsuka, Bunkyo City, Tokyo 112-0012 Japan; 2 Center for Preventive Medical Sciences, Chiba University, 1-33 Yayoi-cho, Inage Ward, Chiba City, Chiba 263-8522 Japan; 3 Teikyo University Graduate School of Public Health, 2-11-1 Kaga, Itabashi City, Tokyo 173-8605 Japan; 4 Department of Preventive Medicine and Public Health, Tokyo Medical University, 6-1-1 Shinjuku, Shinjuku City, Tokyo 160-8402 Japan; 5 Faculty of Nursing, Yamanashi Prefectural University, 1-6-1 Ikeda, Kofu City, Yamanashi 400-0062 Japan; 6 Faculty of Regional Collaboration, Kochi University, 2-5-1 Akebono-cho, Kochi City, Kochi 780-8520 Japan; 7 Faculty of Social Welfare, Nihon Fukushi University, Okuda, Mihama-cho, Chita-gun, Aichi 470-3295 Japan; 8 Center for Gerontology and Social Science, National Center for Geriatrics and Gerontology, 7-430 Morioka-cho, Obu City, Aichi 474-8511 Japan

**Keywords:** Public health, Geriatrics, Lifestyle modification

## Abstract

This study aimed to identify factors associated with engaging in sports and exercise volunteering among older adults. We used cross-sectional data from the Japan Gerontological Evaluation Study (JAGES), a nationwide mail survey of 20,877 older adults from 60 municipalities. Multilevel mixed-effects logistic regression analysis was used to investigate the correlation between engaging in sports and exercise volunteering and 39 variables classified into five factors: (1) demographic and biological, (2) behavioral, (3) psychological, cognitive, and emotional, (4) social and cultural, and (5) environmental factors. Among the analyzed samples, 1580 (7.6%) participants volunteered a few times/year or more often. Factors that showed positive association with the volunteering were older age, a current drinking habit, excellent self-rated health, high proportion of sports group participants in a living area, low municipal population density, and rich social and cultural features (i.e., social cohesion, support, network, and participation). Meanwhile, those that had a negative association were women, low level of education, deteriorated instrumental activities of daily living, having a past or current smoking habit, poor self-rated health, and depressive symptoms. We clarified the characteristics of the population that is more likely to participate in sports and exercise volunteering as well as those of the population that is less likely to participate and requires support.

## Introduction

It is well-known that a proper amount of leisure-time physical activity including sports and exercise is crucial for improving one’s personal health, well-being, and quality of life; there is no exception for older adults^1–3^. Therefore, several studies have explored the factors that promote or hinder the sports and exercise activities of older adults^4–6^. Various factors, including demographic, biological, behavioral, psychological, emotional, social, and environmental aspects have been comprehensively examined.

In recent years, from the perspective of public health and sports promotion, the involvement with sports and exercise other than “playing/doing” has attracted attention. For example, the Second Sport Basic Plan established by The Ministry of Education, Culture, Sports, Science, and Technology, which forms the basis of policies on sports promotion in Japan, encourages people to engage in sports and exercise activities by not only playing but by also “watching” and “supporting” these activities^7^; “supporting” sports and exercise includes volunteer activities (e.g., instruction in exercise and sports, and organizing or supporting sports competitions, events, and club activities)^8^. As the population ages, it is more important to promote “supporting” activities such as volunteering and peer support among older adults as well as rely on an expert instruction and management to disseminate sports and exercise activities more broadly. Several previous studies reported that provision of exercise programs by older volunteers and peers had a positive effect on increasing the physical activity of the participants and spreading the exercise program to the community^9,10^. These benefits to other individuals, combined with the contribution to the healthy aging of the volunteers themselves, encourage volunteer activities in the community, in support of age-friendly cities^11^. In particular, sports and exercise volunteers are expected to acquire a more active lifestyle, since they are more involved in community organizations and less likely to have health problems as barriers to participation, compared to those who participate in general volunteering^12^.

Elucidating relevant factors might be helpful for promoting “supporting” sports and exercise activities (i.e., engaging in sports and exercise volunteering) among older adults. However, most of the prior studies have reported the characteristics of younger adults engaging in volunteer activities in a specific club, sports event, or region^13–15^. In these reports, associations with various factors, including demographic, behavioral, psychological, social, and environmental factors, were examined; the results showed that, for example, being a male, having a higher educational background, and a richer social network are positively associated with engagement in sports and exercise volunteering^13–15^. On the other hand, little is known about the status of participation in sports and exercise volunteers in a population-based sample of older adults and its related factors. In terms of general volunteer activities among older adults (i.e., not limited to sports and exercise), some relevant factors have been identified^16–18^; however, there is no proof that they are similarly applicable since their associations were not consistent in studies of younger adults. As an example, although there are no clear and consistent gender differences with respect to general volunteering^18^, sports and exercise volunteers encompass a notably larger proportion of men^14^. Therefore, this study aimed (1) to clarify the prevalence of sports and exercise volunteering and its specific activities and (2) to identify demographic, biological, behavioral, psychological, emotional, social, and environmental factors associated with engaging in sports and exercise volunteering, using data from a large-scale cohort of older adults in Japan across multiple regions. The findings of the present study provide useful information on the types of sports and exercise volunteer activities that are more approachable for older adults. Furthermore, the study will clarify the characteristics of those who are likely to participate in these volunteer activities, which will suggest avenues for efficient dissemination.

## Methods

### Study design

We used cross-sectional data from the Japan Gerontological Evaluation Study (JAGES), which is an ongoing cohort study exploring social, environmental, and behavioral factors related to the loss of health with regard to functional decline or cognitive impairment among individuals aged ≥ 65 years^19,20^. Between December 2019 and January 2020, we mailed a set of questionnaires to 345,356 community-dwelling people aged ≥ 65 years selected from 60 municipalities including metropolitan, urban, semi urban, and rural communities in 24 prefectures from as far north as Hokkaido (i.e., the northernmost prefecture) and as far south as Kyushu (i.e., the southernmost region) in Japan. A random sample from the official residence registers in 43 large municipalities and a complete census of the older residents of the remaining 17 smaller municipalities were obtained. A total of 240,889 individuals responded to our mail, with a response rate of 69.8% (range, 54.4%–89.8% throughout the 60 municipalities). The questionnaire that included the item of engaging in sports and exercise volunteering was distributed to one-eighth of the participants (n = 29,444) who were randomly selected. We excluded 8567 respondents who did not provide informed consent or information on sex, age, or residence area (n = 5757), had no independence in activities of daily living (n = 2739), or lived in communities with ≤ 30 respondents (n = 71) to avoid non-precise community-level aggregated values due to small samples. In total, we used data from 20,877 eligible respondents (10,123 men and 10,754 women). This data had three levels of hierarchy, with individual-level data (level 1) nested in 1139 community areas defined primarily by the school district (level 2) and then in 60 municipalities (level 3). Within the Japanese context, school districts are the primary residential units of individuals and comprise the geographical settings where older individuals may readily travel via foot or bicycle^21^. Ethical approval for the study was obtained from the Ethics Committee at Chiba University, Japan (Approval number: 2493), the National Center for Geriatrics and Gerontology, Japan (Approval number: 992–3), and the University of Tsukuba, Japan (Approval number: tai020-76). This study was performed in accordance with the principles of the Declaration of Helsinki. All participants were informed that participation in the study was voluntary and that completing the questionnaire, selecting the acceptance checkbox, and returning it via mail indicated their consent to participate in the study.

### Measures

#### Engagement in sports and exercise volunteering

With reference to the survey items of the Japan Sports Agency^8^, participants were queried on their frequency of engaging in sports and exercise volunteering by the following questions: “How often, on average, did you engage in sports and exercise volunteering such as instruction, organizing competitions or events, supporting clubs that you and your family belong to (e.g., transporting participants for practice and competitions, or preparing drinks and lunch) in the past year?” The four possible responses included the following: ≥ 1 day/week, 1–3 days/month, a few times/year, or zero. In addition, we asked those who answered a few times/year or more often about their specific activities from the following options: (1) sports and exercise instruction, (2) sports referee, umpire, and judge, (3) organizing/managing clubs and groups, (4) supporting activities in managing sports facilities, (5) organizing/managing competitions and events, (6) supporting activities in clubs and groups, and (7) other.

#### Selection and categorization of variables

The measures on the questionnaire were selected and classified into the following five categories based on reports of correlates of participation in physical activity among adults^22^ and in sports groups among older adults^6^: (1) demographic and biological, (2) behavioral, (3) psychological, cognitive and emotional, (4) social and cultural, or (5) environmental factors.

#### Demographic and biological factors

We collected the following data as demographic and biological factors: sex, age groups (65–69, 70–74, 75–79, 80–84, ≥ 85 years), marital status (married or unmarried), living alone (no or yes), occupational status (employed, retired, or never employed), and years of education (≥ 13, 10–12, or < 10 years). Annual equivalent income was calculated by dividing household income by the square root of the number of household members and categorized into three groups: ≥ $40,000; $20,000–$39,999; or < $20,000 per year (1 dollar = 100 yen). Disease status was assessed with yes or no answers and included hypertension, stroke, heart diseases, diabetes mellitus, hyperlipidemia, musculoskeletal disorders, and cancer. Body mass index was calculated from self-reported height and weight (kg/m^2^) and categorized as underweight (< 18.5), normal weight (18.5–24.9), or overweight/obese (≥ 25.0). Instrumental activities of daily living (IADL) were assessed using the Tokyo Metropolitan Institute of Gerontology Index of Competence^23^, and the results were classified as good (5 points) or poor (≤ 4 points).

#### Behavioral factors

Alcohol drinking status (none, past, or current) and smoking status (none, past, or current) were collected as behavioral factors.

#### Psychological, cognitive, and emotional factors

We collected the data on self-rated health (excellent, good, poor, or very poor) and divided into three categories (excellent, good, or poor/very poor). We measured depressive symptoms using the short version of the 15-item Geriatric Depression Scale^24,25^ and categorized them into 3 groups: no (0–4 points), mild (5–9 points), or moderate to severe (10–15 points). We rated subjective well-being as 0–10, and binarized 8 points or more as high and 7 points or less as low subjective well-being^26^.

#### Social and cultural factors

We evaluated social and cultural factors in four dimensions: social cohesion (i.e., general trust, norms of reciprocity, and attachment to the neighborhood), social support (i.e., receiving/giving emotional and instrumental social support), social network (i.e., frequency at which participants met with friends, the number of friends met, and interactions with neighbors), and social participation. General trust, norms of reciprocity, and attachment to the neighborhood were categorized as yes (very, moderately) or no (neutral, slightly, not at all)^27^. We dichotomized emotional and instrumental social support, both received and given, as yes or no^27^. Frequency of meeting friends was categorized as almost every day, 2 or 3 times per week, once a week, once or twice per month, or a few times a year or less. We categorized the number of met friends as 0, 1–2, 3–5, 6–9, or ≥ 10. Interactions with neighbors were categorized into 3 groups: cooperating in daily life, standing and chatting frequently, or no more than an exchange of greetings/none. For social participation, we asked respondents about the frequency of participation in hobby activity groups, community associations, senior citizen clubs, study or cultural groups, or activities to teach skills or pass on experiences to others (≥ 1 day/month or < 1 day/month)^27^.

#### Environmental (community- and municipality-level) factors

As community-level (i.e., level 2) factors, the proportion of sports group participants and the perception of park access and sidewalk for each community area was calculated. We defined participating 1 day/month or more as participation in a sports group, and aggregated individual-level sports group participation according to community area^28,29^. In addition, we asked participants if there were parks or sidewalks suitable for exercise or walking within approximately 1 km from their homes, and the percentage of respondents who answered “yes” was calculated by community area. We calculated the population density per km^2^ of inhabitable area and categorized it into a quartile category: ≥ 3,000, 940–2,999, 270–939, and < 270 individuals per km^2^ as a municipality-level (i.e., level 3) factor.

### Statistical analysis

To examine the association between each factor and engaging in sports and exercise volunteering a few times/year or more often, we carried out multilevel mixed-effects logistic regression (the individual as level 1, the community as level 2, and the municipality as level 3) with random intercepts and fixed slopes, which resulted in an odds ratio (OR) and 95% confidence interval (CI) for each variable. Model 1 included demographic and biological, behavioral, psychological, cognitive, and emotional, and environmental factors. We separately added social cohesion, social support, social network, and social participation to model 1 (i.e., model 2, 3, 4, and 5, respectively) because it was assumed that they would strongly correlate with each other. To address potential bias caused by missing values, we adopted multiple imputation under the missing at random assumption (i.e., a missing mechanism is related to the other variables measured in the same survey for that participant). We imputed incomplete variables using a multivariate normal imputation method. We created 20 imputed datasets using all the variables introduced in the current analyses, after which estimated parameters were combined using Rubin’s combination methods^30,31^. We also conducted complete-case analyses as sensitivity analyses. Stata/MP 16.1 (StataCorp, College Station, Texas, USA) was used for all statistical analyses, with P < 0.05 indicating the statistical significance.

## Results

Table 1 summarizes the descriptive data before the implementation of multiple imputation for missing values. Among the 20,877 analyzed participants, 1580 (7.6%) engaged in sports and exercise volunteering a few times/year or more often. Table 2 shows the specific activities of the volunteering they were engaged in. In descending order, it was organizing/managing competitions and events (34.9%), supporting activities in clubs and groups (33.1%), organizing/managing clubs and groups (25.9%), sports and exercise instruction (19.5%), sports referee, umpire, and judge (9.0%), and supporting activities in managing sports facilities (6.9%).

**Table 1:** Descriptive statistics of individual-, community-, and municipality-level variables.

	n	Proportion (%)	Engaging in sports volunteering (a few times a year or more) (%)
***Individual-level variables***			
Total	20,877	100.0	7.6
**Engaging in sports and exercise volunteering**			
Zero	17,284	82.8	
A few times/year	981	4.7	
1–3 days/month	327	1.6	
≥ 1 day/week	272	1.3	
Missing	2,013	9.6	
**Demographic and biological factors**			
**Sex**			
Men	10,123	48.5	9.9
Women	10,754	51.5	5.4
**Age groups (years)**			
65–69	5,232	25.1	7.7
70–74	6,268	30.0	8.0
75–79	5,064	24.3	8.0
80–84	2,910	13.9	6.7
≥ 85	1,403	6.7	5.0
**Marital status**			
Married	15,114	72.4	8.4
Unmarried	5,417	25.9	5.3
Missing	346	1.7	7.5
**Living alone**			
No	17,539	84.0	8.0
Yes	3,085	14.8	5.2
Missing	253	1.2	9.5
**Occupational status**			
Employed	5,839	28.0	9.0
Retired	11,985	57.4	8.0
Never employed	1,128	5.4	4.3
Missing	1,925	9.2	2.6
**Education (years)**			
≥ 13	6,448	30.9	9.1
10–12	8,936	42.8	7.9
< 10	4,892	23.4	4.9
Missing	601	2.9	7.8
**Annual equivalent income**			
≥ $40,000	2,416	11.6	8.7
$20,000–$39,999	7,292	34.9	8.8
< $20,000	8,518	40.8	6.9
Missing	2,651	12.7	5.2
**Disease status (multiple answer)**			
Hypertension	8,960	42.9	7.7
Stroke	467	2.2	6.6
Heart diseases	1,850	8.9	7.2
Diabetes mellitus	2,737	13.1	8.5
Hyperlipidemia	3,038	14.6	7.2
Musculoskeletal disorders	2,036	9.8	5.4
Cancer	847	4.1	6.4
Missing	842	4.0	6.8
**Body mass index**			
< 18.5	1,349	6.5	4.7
18.5–24.9	14,095	67.5	7.7
≥ 25	4,600	22.0	8.0
Missing	833	4.0	7.0
**Instrumental activities of daily living**			
Good	18,771	89.9	7.8
Poor	1,449	6.9	5.5
Missing	657	3.1	6.8
**Behavioral factors**			
**Drinking status**			
None	9,412	45.1	6.1
Past	2,091	10.0	5.5
Current	8,571	41.1	10.0
Missing	803	3.8	4.5
**Smoking status**			
None	12,069	57.8	7.0
Past	6,306	30.2	8.6
Current	2,115	10.1	8.2
Missing	387	1.9	5.4
**Psychological, cognitive, and emotional factors**			
**Self–rated health**			
Excellent	3,081	14.8	11.8
Good	15,288	73.2	7.3
Poor or very poor	2,375	11.4	3.8
Missing	133	0.6	6.0
**Depressive symptoms (Geriatric Depression Scale)**			
No (0–4 points)	15,940	76.4	8.8
Mild (5–9 points)	3,513	16.8	3.8
Moderate to severe (10–15 points)	938	4.5	2.8
Missing	486	2.3	3.5
**Subjective well-being**			
Low (0–7 points)	10,219	48.9	6.3
High (8–10 points)	9,874	47.3	9.0
Missing	784	3.8	5.4
**Social and cultural factors**			
**Social cohesion**			
**General trust**			
No	5,745	27.5	4.7
Yes	14,664	70.2	8.8
Missing	468	2.2	4.3
**Norms of reciprocity**			
No	9,052	43.4	5.6
Yes	11,245	53.9	9.4
Missing	580	2.8	3.3
**Attachment to the neighborhood**			
No	4,079	19.5	4.2
Yes	16,356	78.3	8.5
Missing	442	2.1	4.1
**Social support**			
**Receiving emotional support**			
No	936	4.5	4.2
Yes	19,561	93.7	7.8
Missing	380	1.8	5.0
**Providing emotional support**			
No	1,135	5.4	3.2
Yes	19,217	92.0	7.9
Missing	525	2.5	3.2
**Receiving instrumental support**			
No	1,017	4.9	3.6
Yes	19,518	93.5	7.9
Missing	342	1.6	2.9
**Providing instrumental support**			
No	4,275	20.5	5.2
Yes	15,757	75.5	8.4
Missing	845	4.0	4.5
**Social network**			
**Frequency of meeting friends**			
A few times a year or less	5,575	26.7	2.9
1–3 times/month	4,978	23.8	5.6
1 time/week	2,739	13.1	8.6
2–3 times/week	4,002	19.2	12.4
Almost every day	3,075	14.7	12.8
Missing	508	2.4	3.3
**Number of met friends**			
0	1,726	8.3	1.2
1–2	3,798	18.2	3.1
3–5	5,077	24.3	5.3
6–9	2,761	13.2	7.2
** ≥ **10	6,996	33.5	13.7
Missing	519	2.5	2.5
**Interactions with neighbors**			
Cooperating in daily life	3,520	16.9	11.6
Standing and chatting frequently	11,103	53.2	7.9
No more than exchange greetings/none	5,868	28.1	4.7
Missing	386	1.8	4.4
**Social participation**			
**Hobby activity groups**			
No	10,177	48.7	3.9
Yes	7,928	38.0	13.4
Missing	2,772	13.3	4.6
**Community associations**			
No	10,349	49.6	4.3
Yes	7,790	37.3	13.2
Missing	2,738	13.1	3.9
**Senior citizen club**			
No	15,126	72.5	6.4
Yes	2,931	14.0	17.0
Missing	2,820	13.5	4.1
**Study or cultural groups**			
No	14,889	71.3	6.2
Yes	2,826	13.5	16.9
Missing	3,162	15.1	5.7
**Activities to teach skills or pass on experiences to others**			
No	15,784	75.6	5.6
Yes	2,141	10.3	26.2
Missing	2,952	14.1	4.7
***Community- and municipality-level variables***			
**Environmental factors**			
**Proportion of sports group participants** (community area: n = 1139)			
Mean, standard deviation	24.9	7.7	
**Perception of park access and sidewalk **(community area: n = 1139)			
Mean, standard deviation	81.1	14.2	
**Population density (persons per square km of inhabitable area) by municipality **(n = 60)			
Q1 (≥ 3,000), n	15		
Q2 (940–2,999), n	15		
Q3 (270–939), n	15		
Q4 (< 270), n	15		

Missing values were imputed by using a multivariate normal imputation method in the main regression analysis.

**Table 2:** Specific activities of sports and exercise volunteering (multiple answer).

	Total (n = 1,385)		Men (n = 891)		Women (n = 494)		65–74 years (n = 809)		≥ 75 years (n = 576)	
	n	%	n	%	n	%	n	%	n	%
Organizing/managing competitions and events	484	34.9	340	38.2	144	29.1	295	36.5	189	32.8
Supporting activities in clubs and groups	459	33.1	280	31.4	179	36.2	272	33.6	187	32.5
Organizing/managing clubs and groups	359	25.9	258	29.0	101	20.4	193	23.9	166	28.8
Sports and exercise instruction	270	19.5	192	21.5	78	15.8	157	19.4	113	19.6
Sports referee, umpire, and judge	124	9.0	96	10.8	28	5.7	77	9.5	47	8.2
Supporting activities in managing sports facilities	95	6.9	72	8.1	23	4.7	37	4.6	58	10.1
Other	163	11.8	87	9.8	76	15.4	92	11.4	71	12.3

Proportions (%) were calculated using the number of valid responses for the contents of sports and exercise volunteering as the denominator.

Of the 1,580 respondents who engaged in sports and exercise volunteering, 195 did not respond.

Table 3 shows the ORs for engaging in sports and exercise volunteering according to demographic and biological, behavioral, psychological, cognitive, and emotional, and environmental factors. In the model 1, women, lower level of educational (< 10 years), past and current smoking habit, poor IADL, poor or very poor self-rated health, and mild to severe depressive symptoms were negatively associated with engaging in the volunteering. Age 75 years or older, current drinking habit, excellent self-rated health, higher proportion of sports group participants in a community, and low municipal population density were positively associated with engaging in the volunteering.

**Table 3:** Associations between engaging in sports and exercise volunteering and demographic, biological, behavioral, psychological, cognitive, emotional, and environmental factors.

	**Model 1**		
	OR	95% CI	
***Individual-level variables***			
**Sex (ref. men)**			
Women	0.60	(0.52–0.69)	< 0.001
**Age groups (ref. 65–69** **years)**			
70–74 years	1.16	(1.00–1.35)	0.049
75–79 years	1.38	(1.18–1.62)	< 0.001
80–84 years	1.41	(1.17–1.70)	< 0.001
≥ 85 years	1.38	(1.07–1.78)	0.013
**Marital status (ref. married)**			
Unmarried	0.88	(0.72–1.07)	0.208
**Living alone (ref. no)**			
Yes	0.95	(0.76–1.18)	0.619
**Occupational status (ref. retired)**			
Employed	1.03	(0.92–1.14)	0.654
Never employed	0.94	(0.73–1.21)	0.631
**Education (ref. ≥ 13** **years)**			
10–12 years	0.91	(0.80–1.02)	0.112
< 10 years	0.75	(0.65–0.87)	< 0.001
**Annual equivalent income (ref. ≥ $40,000)**			
$20,000–$39,999	1.07	(0.91–1.26)	0.387
< $20,000	1.09	(0.93–1.28)	0.269
**Disease status (ref. none of each)**			
Hypertension	1.03	(0.92–1.14)	0.609
Stroke	0.92	(0.63–1.34)	0.670
Heart diseases	0.93	(0.79–1.10)	0.421
Diabetes mellitus	1.15	(0.98–1.34)	0.089
Hyperlipidemia	0.91	(0.78–1.06)	0.211
Musculoskeletal disorders	1.01	(0.81–1.26)	0.918
Cancer	0.90	(0.68–1.19)	0.456
**Body mass index (ref. 18.5–24.9)**			
< 18.5	0.83	(0.66–1.05)	0.122
≥ 25	1.05	(0.92–1.20)	0.482
**Instrumental activities of daily living (ref. good)**			
Poor	0.67	(0.53–0.84)	0.001
**Drinking status (ref. none)**			
Past	0.87	(0.71–1.07)	0.176
Current	1.23	(1.06–1.43)	0.006
**Smoking status (ref. none)**			
Past	0.78	(0.67–0.91)	0.002
Current	0.81	(0.66–0.99)	0.044
**Self-rated health (ref. good)**			
Poor or very poor	0.70	(0.57–0.87)	0.001
Excellent	1.49	(1.31–1.71)	< 0.001
**Depressive symptoms (ref. no)**			
Mild	0.62	(0.51–0.75)	< 0.001
Moderate to severe	0.56	(0.38–0.83)	0.004
**Subjective well-being (ref. low, 0–7)**			
High, 8–10	1.11	(0.98–1.26)	0.108
***Community- and municipality-level variables***			
**Proportion of sports group participants**			
Per 10 percentage points	1.12	(1.04–1.21)	0.004
**Perception of park access and sidewalk**			
Per 10 percentage points	0.97	(0.92–1.02)	0.283
**Population density (persons per square km of inhabitable area) (ref. the highest quartile, Q1)**			
Q2	1.25	(1.06–1.48)	0.009
Q3	1.78	(1.46–2.15)	< 0.001
Q4	1.58	(1.22–2.05)	< 0.001

n = 20,877.

All variables shown were simultaneously added in the model.

*OR* odds ratio, *CI* confidence interval.

Table 4 shows the results of models 2–5 with social cohesion, social support, social network, and social participation added to model 1, respectively. All variables except for receiving emotional and instrumental support were positively associated with engaging in sports and exercise volunteering.

**Table 4:** Associations between engaging in sports and exercise volunteering and social cohesion, support, network, and participation.

	OR	95% CI	*P*
**Model 2: model 1 + social cohesion (ref. none of each)**			
General trust, yes	1.16	(1.01–1.33)	0.038
Norms of reciprocity, yes	1.27	(1.11–1.44)	< 0.001
Attachment to the neighborhood, yes	1.25	(1.05–1.49)	0.014
**Model 3: model 1 + social support (ref. none of each)**			
Receiving emotional support, yes	1.10	(0.77–1.56)	0.609
Providing emotional support, yes	1.50	(1.07–2.11)	0.019
Receiving instrumental support, yes	1.26	(0.90–1.78)	0.175
Providing instrumental support, yes	1.33	(1.14–1.54)	< 0.001
**Model 4: model 1 + social network**			
Frequency of meeting friends (ref. a few times a year or less)			
1–3 times/month	1.28	(1.05–1.55)	0.014
1 time/week	1.80	(1.48–2.19)	< 0.001
2–3 times/week	2.35	(1.94–2.84)	< 0.001
Almost every day	2.08	(1.68–2.57)	< 0.001
Number of met friends (ref. zero)			
1–2	1.57	(1.03–2.37)	0.034
3–5	1.87	(1.25–2.81)	0.003
6–9	2.11	(1.39–3.18)	< 0.001
≥ 10	3.33	(2.25–4.93)	< 0.001
Interactions with neighbors (ref. no more than exchange greetings/none)			
Standing and chatting frequently	1.32	(1.14–1.52)	< 0.001
Cooperating in daily life	1.75	(1.49–2.06)	< 0.001
**Model 5: model 1 + social participation (ref. none of each)**			
Hobby activity groups, yes	2.00	(1.75–2.29)	< 0.001
Community associations, yes	1.71	(1.49–1.97)	< 0.001
Senior citizen club, yes	1.60	(1.39–1.83)	< 0.001
Study or cultural groups, yes	1.20	(1.04–1.38)	0.012
Activities to teach skills or pass on experiences to others, yes	2.81	(2.44–3.24)	< 0.001

n = 20,877.

All variables shown were simultaneously added in each corresponding model.

*OR* odds ratio, *CI* confidence interval.

Supplementary Tables 1 and 2 show the results of the complete-case analysis for each model. Among the analyzed participants, data from 11,854 (56.8%) individuals had no missing values. Of the 9,023 participants with missing values, the mean number of missing values was 4.1. The complete-case analyzes produced similar results to those of the multiple-imputation analyzes, other than that the positive association in the 80 + age category disappeared.

## Discussion

To the best of our knowledge, this is the first study that examines the current state of sports and exercise volunteering among older adults and that comprehensively elucidates its correlates by using data from a large population-based sample. We found that 7.6% of older adults engaged in sports and exercise volunteering once a year or more often, and the main activities were organizing, managing, or supporting competitions, events, clubs, or groups, and instruction. Further, we clarified various demographic, biological, behavioral, psychological, cognitive, emotional, social, cultural, and environmental factors related to the activity. Although there have been several reports of the characteristics of older adults who participate in sports and exercise groups as well as general volunteer activities, the present study provides new insights specific to sports and exercise volunteering.

In the survey conducted by the Japan Sports Agency^8^, which was the basis of the volunteering items in the present study, 5.3% of respondents in their 60’s and 5.2% of those in their 70’s engaged in sports and exercise volunteering within a year. Among these, the specific activities of each age group (60’s and 70’s) were as follows: organizing/managing competitions and events (31.4% and 29.1%), exercise and sports instruction (25.4% and 20.6%), and organizing/managing sports clubs and groups (19.5% and 24.8%)^8^; the results found in the present study approximately corroborated these reports.

We confirmed that participants with advanced age engaged more often, and women had less engagement in sports and exercise volunteering. In general, it has been shown that the frequency of participation in sports groups among older men with more advanced age was low compared to older women with younger age^6^; however, confirming an opposite association in the present study was noteworthy. In addition, age and volunteer activities have been reported to be negatively^32^ or not correlaed^16,17^; the findings of this study are also inconsistent with these previous reports. To conduct volunteer activities related to sports and exercise (e.g., organizing/managing competitions, events, clubs, and instructing/refereeing sports), participants would need to have a certain experience with the sports and exercise. It is speculated that if they become veterans by continuing to participate in sports and exercise activities, they will play various leading and management roles. In particular, men may tend to play such roles, partly because they have more administrative job experience than women during their active career^33^.

Participants with lower levels of education were less likely to engage in sports and exercise volunteering. In Western countries, there is a positive association between volunteer activities at an older age and educational attainment^18,34^, whereas no significant association has been observed in Japan^17^. Namely, in the Japanese context, the present results may be specific to sports and exercise volunteering. As for sports and exercise group participation, in Japan, the participation rate is lower among older adults with lower education levels^6^, and this aspect may have affected the results of the current study. According to the other demographic and biological factors and psychological, cognitive, and emotional factors, the findings of a negative association between poor IADL, having depressive symptoms and a positive association between excellent self-rated health were mostly similar to the findings of prior studies on the correlates of sports and physical activities^6,35,36^ or volunteer activities^17,37^ among older adults; these factors are commonly associated with activities related to sports, exercise, and volunteering among older adults.

Regarding behavioral factors, current alcohol drinking habits were positively associated, and smoking habits in the past and current were negatively associated with sports and exercise volunteer engagement. In a systematic review of adults that summarized correlates associated with physical activity, drinking and smoking habits were reported to be inconclusive^22^. Conversely, according to a report that comprehensively examined the factors related to sports group participation in older adults in Japan, similar to the findings of the present study, drinking habits showed a positive association and smoking habits showed a negative association^6^. Participation in sports or volunteer groups naturally involves interaction with others, unlike individual physical activity; therefore, a link to these behavioral factors may obviously emerge. Especially in recent years in Japan, smoking in public places has been strictly controlled to prevent involuntary or passive smoking. This may keep smokers away from participating in public activities.

As for environmental factors, municipality-level low population density and high proportion of community-level sports group participation showed a positive relationship with the engagement of sports and exercise volunteering. A previous study comparing the social participation among older adults in Japan between urban and rural areas confirmed that rural areas have more volunteer group participants and less sports group participants than urban areas^38^. There is a possibility that people tend to participate in sports and exercise by utilizing public and private services and facilities in urban areas; while in non-urban areas, they are likely to organize and manage sports and exercise groups themselves especially in active area of sports group participation.

Fertile social and cultural factors (i.e., social cohesion, support, network, and participation) were widely positively associated with engagement of sports and exercise volunteering. The present findings partially supported the results of earlier studies that investigated correlates of volunteer participation among older adults, not limited to sports and exercise volunteering^17,39^. These studies reported that older adults engaging in volunteer activities had a higher level of community interactions evaluated from the aspect of community involvement and satisfaction, and more often participated in hobby and continuing education activities compared to those who did not engage in. Volunteering is often motivated by interacting with friends and gaining the approval of others considered to be important by addressing community concerns and social needs, rather than monetary rewards^40^. Therefore, social cohesion, support, network, and participation could be considered a non-monetary motivation and benefit for engagement of sports and exercise volunteering. Furthermore, sports and exercise volunteering may also provide potential physical benefits, such as the inclusion of a physical activity component within the activity itself and more frequent time outside of the house due to broader social participation and social networks.

The strength of the present study was its large, nationwide, and population-based sample enabling municipality-, community-, and individual-level multilevel analyses for elucidating the correlates of sports and exercise volunteering among older adults. However, some limitations of this study warrant discussion. First, we could not infer a causal relationship because of the nature of the cross-sectional design; further longitudinal studies are required to address this limitation. Second, regarding behavioral and environmental factors, relationships with only a few items were investigated. Furthermore, environmental factors were mainly variables based on self-report and perception, and it was not possible to fully consider the built environment based on objective evaluation. Future studies should use geographic information systems to collect more detailed information. Third, external validity of the findings obtained in this study could not be discussed. Different relationship could be found, especially in Western countries where the culture, manners, and customs of sports and volunteering are different from those in Japan. In addition, we cannot ignore sampling bias due to the non-response of approximately 30% of the invited individuals, and selection bias due to the choice of analytic participants who were independent in their daily living activities; together, these factors may have biased the results toward those with a higher health status. Therefore, the proportion of volunteers may have been overestimated, and the association with each factor may have been either over- or underestimated. Fourth, there was a different association with age in the multiple-imputation and complete-case analysis. As such, the finding that adults aged 80 years and older were more likely to engage in sports and exercise volunteering should be interpreted with caution.

## Conclusion

We found that 7.6% of the population-based sample of older adults in Japan engaged in sports and exercise volunteering such as organizing, managing, or supporting competitions, events, clubs, or groups, and instruction. We clarified factors that show a positive association with the activity (i.e., high age, having current drinking habit, excellent self-rated health, high proportion of sports group participants in a living area, low municipal population density, and rich social and cultural features) and those that show a negative association (i.e., women, low educational attainment, deteriorated IADL, having past or current smoking habit, poor self-rated health, and depressive symptoms). We equally characterized the population that is more likely to participate in sports and exercise volunteering and the population that is less likely to participate and requires support, and provided clues for strategic dissemination of “supporting” sports and exercise. Recruiting volunteers from the populations that are more likely to participate at first, and then involving more people by making good use of their social networks and support might be an effective strategy.

## Supplementary Information

Supplementary Table 1.

Supplementary Table 2.

## Data Availability

The data underlying this study is from the JAGES and contain sensitive information. Data for research purposes is available upon request. Requests for the JAGES data can be made to dataadmin.ml@jages.net.
